# Experiences of LGBTI+ individuals in accessing right to health; a survey study from Turkey

**DOI:** 10.4314/ahs.v22i4.38

**Published:** 2022-12

**Authors:** Meltem Gunbegi, Elif Sazak Uygul, Ahmet Turla, Berna Aydin

**Affiliations:** Department of Forensic Medicine, Faculty of Medicine, Ondokuz Mayis University, Atakum/SAMSUN, 55139-Turkey

**Keywords:** LGBTI+, health service, right to health, discrimination, access to health services

## Abstract

**Background:**

In Turkey, LGBTI+s is experiencing discrimination in all areas of their lives.

**Objectives:**

We aimed to determine the problems experienced by LGBTI+s living in Turkey in accessing their right to health.

**Methods:**

An online questionnaire containing 37 open-ended and multiple-choice questions about respondent's demographic characteristics, experiences in accessing and receiving health services was prepared. Between October 2018 and December 2019, the survey was disseminated via social media platforms and sent to LGBTI+ friendly institutions. 81 people responded to the survey.

**Results:**

43.2% had at least one chronic disease. Participants stated that 44.5% of them go to a health institution <3 times in a year. 91.4% of the participants declared that they never or rarely said their sexual identity/orientation at the health institutions, and 39.2% of them encountered negative behaviour when they did. 98.8% of the participants said that they think physicians do not have enough information about LGBTI+s.

**Conclusions:**

The knowledge and attitude of healthcare professionals are one of the essential determinants of LGBTI+s' use of their right to health. Alienating and homophobic behaviors against LGBTI+s is the biggest problem for LGBTI+s to receive quality health care in Turkey.

## Introduction

Despite the absence of definitive data on individuals' sexual orientation due to the lack of regular records and *“social sensitivities”* not yet left behind, it is estimated that the share of lesbian, gay, bisexual, transsexual and intersex individuals and those with other sexual orientation, shortly known as LGBTI+, in world population varies in the range 5 to 10%. While this share is already high, according to the 2017 report of the European Commission. 1 LGBTI+ individuals experience problems more frequently than heterosexual individuals in their access to health services and communication with healthcare personnel. The same report also identifies the insufficiency of studies dealing with access to healthcare and level of health literacy of LGBTI+ individuals, especially trans and intersex individuals, in particular as a common and important problem.^2^

Relating to the right to health of LGBTI+, both national and international legislation include many provisions banning discrimination on grounds of an individual's “sexual orientation and sexual identity”. Article 25 in the Universal Declaration of Human Rights states that *“Everyone has the right to a standard of living adequate for the health and well-being of himself and of his family, including food, clothing, housing and medical care and necessary social services, and the right to security in the event of unemployment, sickness, disability, widowhood, old age or other lack of livelihood in circumstances beyond his control.”*^3^

According to the Recommendation no. 5 by the Committee of Ministers of the Council of Europe where Turkey is a member state on measures to combat discrimination on grounds of sexual orientation or gender identity, “Member states should take appropriate legislative and other measures to ensure that that the highest attainable standard of health can be effectively enjoyed without discrimination on grounds of sexual orientation or gender identity; in particular, they should take into account the specific needs of lesbian, gay, bisexual and transgender persons in the development of national health plans including suicide prevention measures, health surveys, medical curricula, training courses and materials, and when monitoring and evaluating the quality of healthcare services”.^4^

While conventions and legislative arrangements explicitly guarantee equal rights for all and qualify gender-based discrimination as an offence, relevant literature cites many cases where LGBTI+ individuals face gender-based discrimination in their social lives and public sphere.^3^,^5^–^12^ The Sexual Orientation and Sexual Identity Based Human Rights Monitoring Report covering the period 2013–2017 mentions cases where LGBTI+ individuals experience problems with respect to their rights and face discrimination. 13 There are many publications reporting that besides facing discrimination and hate crimes LGBTI+ individuals also have problems in service delivery and even are unable to receive quality services.^2^,^14^,^15^ Avoidance in declaring gender identity to healthcare providers, discrimination faced when it is declared and resulting hesitation to visit health institutions are also problems that are reported. 16,17

Data relating to cases of discrimination that LGBTI+ face in exercising their right to health in Turkey are mostly derived from media news or sharing of LGBTI+ friendly organizations while there is shortage of scientific data and official records. Social policy studies covering such areas as employment, housing, health services, social services, care and education are relatively scarce.^18^The absence of sufficient data and information on the existing problems is one of the most fundamental problems standing on the way to the adoption of legal and other measures to prevent discrimination. Capitalizing on this fact, the present study seeks to expose and evaluate problems that LGBTI+ in Turkey face while receiving health services.

## Methods

For this work planned as a descriptive survey, an online questionnaire with 37 questions was developed over Google Forms. Questions were formulated by considering earlier studies in the field and reported cases of violation of rights. The first part of the questionnaire has 7 questions related to age, city of residence, monthly income, health insurance coverage, and demographic characteristics in the context of gender and sexual orientation. The second part includes open-ended and multiple-choice questions on participants' experiences in access to preventive and curative services and their personal experiences with physicians. In the introductory part of the questionnaire, there is warning that personal information including full name and identity number should be given in no part of the document and a note saying responses given by voluntary participation will be used solely for scientific purposes. And those who gave their consent participated to the survey.

To identify problems that may be confronted in the application of the survey and to check whether questions are understandable, the draft questionnaire was first administered with 5 persons and then finalized after necessary modifications. The final form was then disseminated through social media platforms and sent to LGBTI+ friendly organizations in the period 15 October 2018–20 December 2019.

81 persons responded to the questionnaire that was kept active on the portal until 20 December 2019. Data obtained was evaluated by using SPSS 21.0 programme. Descriptive statistics were used for data analysis. The study was performed according to the Helsinki Declaration Ethical principles.

## Results

**Figure 1:**
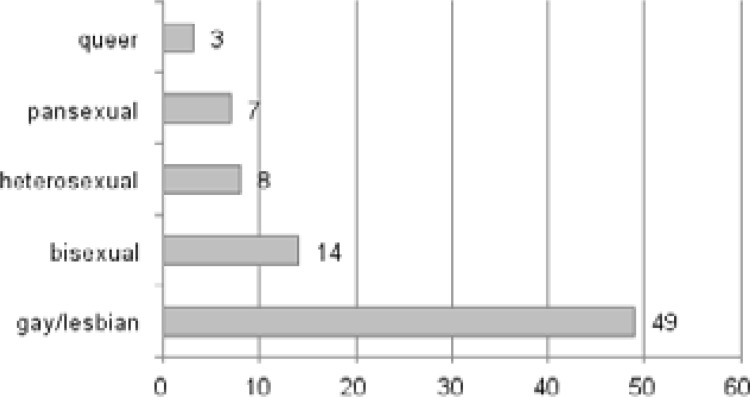
Distribution of Participants by their Sexual Orientation.

The average age of survey participants was 28.6±7,05 (min:18 - max:55). Out of 50 participants responding to the question, 21 stated their gender identity as “male”, 13 as “female”, 13 as “trans” and 3 as “intersex”. Responses to the question on sexual orientation showed that 60.5% (n:49) were “gay/lesbian” The distribution of sexual orientation is given in Figure 1.

**Figure 2:**
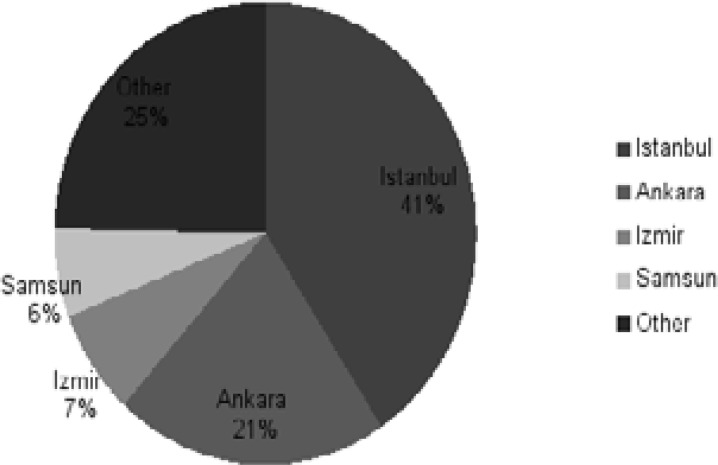
Distribution of Participants by Provinces They Live.

It was observed that participants mainly live in big cities like Istanbul (41%) and Ankara (21%) while all geographical regions of the country were represented (Figure 2).

**Figure 3:**
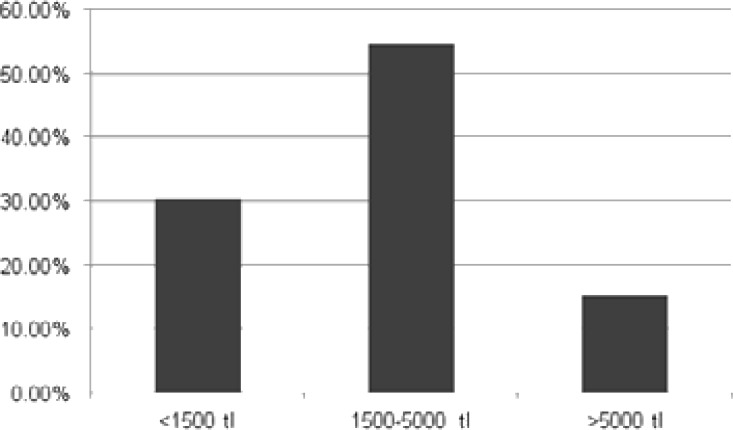
Distribution of Patients by their Levels of Monthly Income.

While monthly minimum wage was 2020 TL (Turkish Lira) (359 $ (US Dollars)) in Turkey in 2019, 30.4% of 79 participants responding to the question on monthly income had their monthly income of less than 1,500 TL (267 $). Figure 3 gives monthly income figures of participants. 85.2% of participants were covered by health insurance scheme and 14.8% had no health insurance.

**Figure 4:**
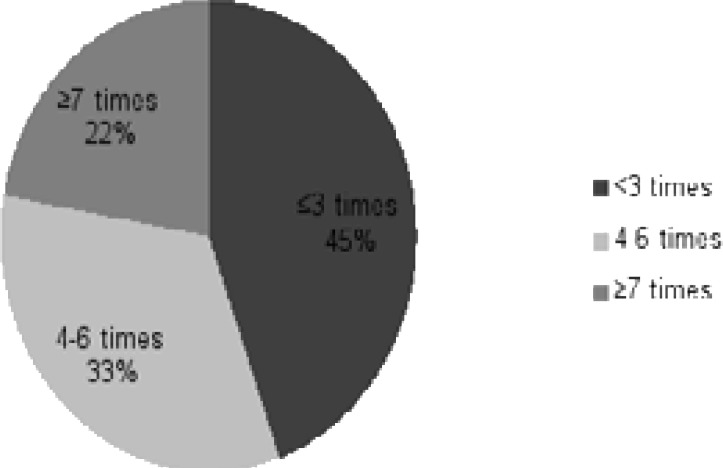
Distribution of Patients by their Number of Annual Applications to Health Institutions.

In response to the question on frequency of application to a health institution in a given year, 44.5% said 0–3 times and 33.3% 4–6 times. 43.2% of participants had chronic illnesses (Figure 4).

52.3% of the participants stated that they usually go to outpatient clinics when they need to use health services, and 25% of the participants go to the emergency department. The rest of them prefer family medicine clinics.

29.6% of participants had their preference for private hospitals and private physicians while 70.4% preferred state and university hospitals. 28.8% of participants said they prefer the health institution according to their gender identity or sexual orientation.

87.7% of the participants did not pass a national screening program (breast cancer, cervical cancer, HPV screening, colorectal cancer screening, etc.), and 73% of them stated that they wanted to have a screening test.

18 participants responded to the open-ended question *“How does your gender identity or sexual orientation influence your preference”* related to the selection of health institution to apply. 5 participants said they made their preference depending upon the attitude of health workers, while 3 preferred private health institutions and 4 preferred university hospitals. There were also other responses mentioning such factors as educational background and institution of the physician concerned, preference for female physicians, preference for an institution where he/she will remain anonymous and by referring to physicians who are not heterosexist and transphobic.

44 participants responded to the open-ended question *“How does your gender identity and orientation affect your selection of physicians?”*. According to these responses, the attitude and approach of physicians make a difference in this regard. Besides, recommendation by friends and comments on the internet are also taken into account together with information about the competence, level of information and experience of professionals, their educational background and being a female as a factor for preference. Here are two responses to the question: *“I choose my gynaecologist for not being discriminatory and others on the basis of their work in their fields” and “I visit a psychiatrist who would not give any transphobic reaction when I declare my sexual identity”*.

66.7% of participants have worries that they may face discrimination due to their gender identity or orientation when they apply to a health institution and 52.9% of them stated that they avoid applying to a health institution.

39.2% of respondents declared that when they reveal their gender identity or sexual orientation, the health personnel's attitude most of the time or always changes negatively toward them.

25 responses were given to the open-ended question *“Can you explain how and in which way you face discrimination while receiving health services”*. While 12 respondents mentioned “disturbing looks” others referred to verbal and behavioural acts of discrimination.

63.3% of respondents (50 out of 79) declared that they couldn't receive the same quality health service as heterosexuals. 40 participants responded to open-ended question *“Why can't you receive the same service as heterosexuals?”*. The most common response was that they cannot receive adequate health service because they feel obliged to hide their identity. Other explanations include discrimination, homophobia/transphobia and heteronormatively structured medicine and order. Further, there were also comments like *“I cannot enjoy the same service, because I cannot apply to any physician; I don't think I am guided correctly since I cannot mention all forms of relationship I am engaged in; I cannot receive the same service in gynaecological exams and our trans friends cannot receive equal services”; “Not me, but our transgender friends are highly complainant on this”; and “Doctors are not sufficiently informed about trans identities and approach these matters mostly on the basis of some myths.”*

91.4% of respondents said they never or rarely declare their sexual identity/orientation in their applications. 79 persons responded to the question *“Do you come across any negative behaviour when you specify your sexual identity and/or orientation?”* of whom 39.2% said “often and all the time”, 49.4% said “rarely” and 11.4% **“never”**.

47.4% of respondents (37 out of 78) think the gender of the group of physicians is a factor influencing their discriminatory attitude and 80.6% (29 out of 36) of them think especially male personnel have more discriminatory attitudes than females. 43.6% (34 out of 78) say it is the age of physicians that is influential in this regard, and 76,5% (26 out of 34) of them think below the age of 55 years old physicians have more discriminatory attitudes. 98.8% of respondents think that physicians are not sufficiently informed about LGBTI+ individuals.

96.3% of participants said they would like to have training for LGBTI+ in methods of protection from sexually transmitted diseases. Responses by participants to other questions are given in Table 1.

**Table 1:** Distribution of Responses Given by Participants to Some Questions

	QUESTIONS	YES		NO		TOTAL	
		n	*%*	n	*%*	n	*%*
**1**	Do you have your health insurance?	69	*85.2*	12	*14.8*	81	*100*
**2**	Have you passed through any national screening programme before? (i.e., breast cancer, prostate cancer, cervix cancer - HPV screening...)	10	*12.3*	71	*87.7*	81	*100*
**3**	Do you specifically select the physician that you will receive health services from?	45	*55.6*	36	*44.4*	81	*100*
**4**	Do you have worries that you may face discrimination due to your gender identity or orientation when you apply to a health institution?	54	*66.7*	27	*33.3*	81	*100*
**5**	Do you think it is necessary to tell health personnel about your gender identity or orientation in each application?	7	*8.6*	74	*91.4*	81	*100*
**6**	Do you think it is necessary for the health personnel to ask your gender identity or orientation in each application?	11	*13.6*	70	*86.4*	81	*100*
**7**	In your hospital applications have you faced discrimination on grounds of your gender identity or orientation?	32	*39.5*	49	*60.5*	81	*100*
**8**	Do you encounter sexist discourses while receiving health service?	46	*56.8*	35	*43.2*	81	*100*
**9**	Do you think health services you receive are of same nature and quality as heterosexual individuals?	29	*36.7*	50	*63.3*	79	*100*
**10**	Do you think you receive/can receive adequate information from health institutions about methods of protection?	20	*25*	60	*75*	80	*100*

## Discussion

Since general population censuses and studies in Turkey question gender characteristics only by male-female” distinction and there is no demographic survey focusing on sexual orientation, it is quite difficult to give a percentage of LGBTI+ individuals in total population. Given this, it is also difficult to assess to what extent our present study reflects the actual situation.

28.6 as the average age of participants to our survey can be explained by the fact that social media is used more widely by younger generations. The study found the percentage of participants covered by health insurance scheme quite high (85.2%). The percentage of others who are not covered by any health insurance scheme (14.8%), which is one of the fundamental rights guaranteeing access to health services, is similar to the finding of another and wider-scale survey.^17^ However, a study conducted in Istanbul in 2010 with 116 trans women found that 79.3% had no health insurance.^19^ This is quite high relative to the figure we obtained in our study. This difference is possibly due to limited participation of trans individuals who constitute the most insecure part of LGBTI+ to our study.

While the correctness of quantitative values relating to income levels of individuals in the country is an important issue under debate, our survey found the share of persons at low- and medium-income levels high. It is known that LGBTI+ individuals can be found in all professional groups and findings about their income status are similar to those found by other studies.^17^,^20^ Mobbing in the process of job recruitment in particular and later during employment are among reported cases of discrimination. 18 Low level of income is one of the important reasons limiting access to healthcare services.^21^ It is therefore important to take special steps in relation to disadvantaged groups.

The survey found the higher incidence of application to public hospitals (70.4%). This finding too is in line with the findings of other similar studies.^16^,^22^ Despite the recent increase in the number of private health institutions, the weight of the public in this regard still persists.

Though participants were from a younger age group, 43.2% were found to have their chronic diseases which is also similar to the findings of other studies.^22^ Consequently, their frequency in application to health institutions is quite high. Recent studies suggest that major health problems of LGBTI+ individuals include psychological problems, problems deriving from smoking and higher risk of cancer.^23^,^24^ In spite of these risks, the rate of those who are not covered by any national screening programme is quite high (87.7%). These outcomes suggest that scientific studies identifying special risk factors for LGBTI+ are yet insufficient and that there are gaps in the delivery of protective healthcare for existing risks.^25^

Again, in accord with other studies, our survey found high rate of concern for the possibility of facing discrimination in application to health institutions.^21^This high concern manifests itself in unwillingness in declaring sexual orientation or objection to the posing of this question by physicians.

It appears that the physician who is to deliver healthcare service is a determining factor in the selection of health institution to apply by LGBTI+ individuals.26 Both our survey and other similar ones show that the age and gender of the physician are also factors influential on selection. 22Here, higher preference for female physicians cannot be considered as separate from gender roles. Recommendation by other LGBTI+ persons, online comments by patients, attitudes of physicians in earlier applications, gender (females are preferred), level of education and information of physicians are influential in selections while patients act more selectively in deciding for their gynaecologists and psychiatrists. Short responses indicating these are explanative of worries about discrimination while applying to health institutions.^26^

The rate of facing discrimination while receiving health services found as 39.5% in our survey is compatible with some studies on the same issue and quite low compared to some others^16^,^20^,^22^,^27^ This difference may be attributed to the low number of trans participants to our survey and to the fact that other participants do not declare their identities in their applications. It is normally assumed that LGBTI+ individuals who make their identity open or whose identity can be inferred from their appearance, do face serious cases of discrimination in the field of health as well. This idea is confirmed by the fact that a quite high percentage of participants (91.4%) never or rarely declare their gender identity/sexual orientation in their applications and they say they mostly face negative behaviour when they declare it.

The acts of discrimination are mainly allusive attitude and behaviour including ways of looking, use of different discourse and some acts as described similarly in other studies.^23^,^26^ The study by Albuquerque et. al. stresses that health workers do display discriminatory, prejudiced and stigmatizing attitudes against LGBTI+ individuals.^28^ Another study investigating obstacles that LGBTI+ face in the process of healthcare services states that prejudices by health workers is one of the factors affecting their access to quality services.^29^ The findings we obtained from our survey, which are in conformity with other similar studies, suggest that health workers do not always act in compliance with the ethical rules of the profession and display discriminatory behaviour although gender/sexual orientation-based discrimination is an offence. It is considered that educations for tolerance to differences, professional responsibility and related legal sanctions may be functional in preventing an important part of this discriminatory behaviour.

Concerns reflecting the opinion of participants that they cannot receive services on equal footing with heterosexuals must be seriously taken into account: *“I cannot enjoy the same service, because I cannot apply to any physician; I don't think I am guided correctly since I cannot mention all forms of relationship I am engaged in; I cannot receive the same service in gynaecological exams and our trans friends cannot receive equal services”*. There are also other studies indicating the conviction of LGBTI+ individuals that they cannot receive health services on an equal footing with the rest of population.^20^,^22^

The finding of the survey that almost all participants (98.8%) consider physicians as insufficiently informed about LGBTI + individuals and their health is in compliance with the findings of other surveys in the same field.^22^,^25^ This lack of information on the part of health service providers concerning the health status of LGBTI+ derives from the fact that the science of medicine is shaped exclusively on the basis of heterosexuals and that medical education does not cover LGBTI+. Besides insufficient information, existing inequalities may also be attributed to the attitude of health service providers that is based not on ethical rules but their own beliefs and moral codes.

## Conclusion

This study tried to identify the problems of LGBTI+ in Turkey in their access to and utilization of health services based on a small percentage of these individuals. A common finding of studies in this field is that the level of information and attitude of physicians is an important determinant in access to the right to health. Attitudes that otherize LGBTI+, of homophobic nature and even constituting hate crime block LGBTI+ individuals' proper enjoyment of healthcare services. The otherizing discourse by the media, anti-LGBTI+ stance of politicians and top-level managers, and biased education delivered starting from early years of schooling up to university are factors contributing to homophobia among health workers who are delivering a public service. Therefore, it must be kept in mind that it would be an important step for the solution to the problem of LGBTI access to the right to quality health if the education and training of physicians cover the issue of LGBTI health.

## Limitation

In this study, snowball sampling method was used. In this method the survey is directed by the participants to other participants. As a result of the snowball sampling method, only 81 people participated in the survey due to number of participants being limited to the participants and the people they could reach.
